# Characterizations of Alpha-Cellulose and Microcrystalline Cellulose Isolated from Cocoa Pod Husk as a Potential Pharmaceutical Excipient

**DOI:** 10.3390/ma15175992

**Published:** 2022-08-30

**Authors:** Olutayo A. Adeleye, Oluyemisi A. Bamiro, Doha A. Albalawi, Amenah S. Alotaibi, Haroon Iqbal, Saheed Sanyaolu, Mbang N. Femi-Oyewo, Kehinde O. Sodeinde, Zwanden S. Yahaya, Gobika Thiripuranathar, Farid Menaa

**Affiliations:** 1 Department of Pharmaceutics and Pharmaceutical Technology, Federal University Oye-Ekiti, Oye-Ekiti 3600001, Ekiti State, Nigeria; 2 Department of Pharmaceutics and Pharmaceutical Technology, College of Pharmacy, Afe Babalola University, Ado-Ekiti 360101, Ekiti State, Nigeria; 3 Department of Pharmaceutics and Pharmaceutical Technology, Olabisi Onabanjo University, Ago-Iwoye 120107, Ogun State, Nigeria; 4 Department of Biology, Faculty of Science, University of Tabuk, Tabuk 71491, Saudi Arabia; 5 Genomic & Biotechnology Unit, Department of Biology, Faculty of Science, University of Tabuk, Tabuk 71491, Saudi Arabia; 6 The Cancer Hospital of the University of Chinese Academy of Sciences (Zhejiang Cancer Hospital), Institute of Basic Medicine and Cancer (IBMC), Chinese Academy of Sciences, Hangzhou 310022, China; 7 Department of Industrial Chemistry, Federal University Oye-Ekiti, Oye-Ekiti 3600001, Ekiti State, Nigeria; 8 Department of Pharmaceutics and Industrial Pharmacy, Kaduna State University, Kabala Coastain 800283, Kaduna State, Nigeria; 9 Institute of Chemistry Ceylon, College of Chemical Sciences, Rajagiriya 10107, Sri Lanka; 10 Department of Pharmaceutics and Nanomedicine, Fluorotronics Inc. and California Innovations Corp., San Diego, CA 92037, USA

**Keywords:** alpha cellulose, microcrystalline cellulose, cocoa pod husk, direct compression, metronidazole tablet

## Abstract

Cellulose is a non-toxic, bio-degradable, and renewable biopolymer which is abundantly available in nature. The most common source of commercial microcrystalline cellulose is fibrous wood pulp. Cellulose and its derivatives have found wide commercial applications in the pharmaceutical, cosmetic, food, paper, textile, and engineering industries. This study aims to isolate and characterize cellulose forms from cocoa pod husk (CPH) and to assess its mechanical and disintegration properties as a direct compression excipient in metronidazole tablets. Two isolated cellulose types (i.e., cocoa alpha-cellulose (CAC) and cocoa microcrystalline cellulose (C-MCC)) were compared with avicel (AV). CAC and C-MCC were characterized for their physicochemical properties using Scanning Electron Microscopy (SEM), FTIR spectroscopy, Differential Scanning Calorimetry (DSC), and X-Ray Powder Diffraction (XRD). Metronidazole tablets were produced by direct compression with cellulose. The mechanical and disintegration properties of the tablets were evaluated. CAC and C-MCC yield was 42.3% *w*/*w* and 38.25% *w*/*w*, respectively. Particle diameters were significantly different with CAC (282.22 μm) > C-MCC (161.32 μm) > AV (72.51 μm). CAC and C-MCC had a better flow than AV. SEM revealed the fibrous nature of the cellulose. FTIR and XRD analysis confirmed the presence of cellulose with crystallinity index of 69.26%, 43.83%, and 26.32% for AV, C-MCC, and CAC, respectively. C-MCC and AV are more crystalline and thermally stable at high temperatures compared to CAC. The mechanical and disintegration properties of C-MCC and AV tablets complied with pharmacopeia specifications. Taken together, C-MCC isolated from CPH displayed some fundamental characteristics suitable for use as a pharmaceutical excipient and displayed better properties compared to that of AV.

## 1. Introduction

**Figure 1:**
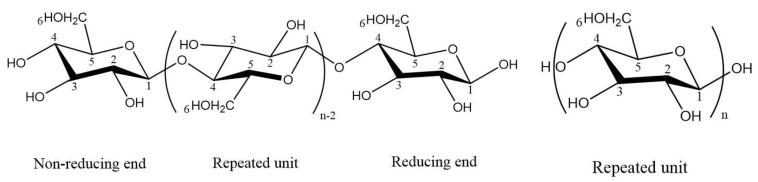
Chemical structure of cellulose (C_6_H_10_O_5_)_n_. ChemDraw Pro 8.0 was used to represent this linear homopolymer composed of repeated units of AUG linked together by *β*-(1-4)-glycosidic bonds.

Cellulose is a non-toxic, bio-degradable, and renewable biopolymer derived from biomass, and so, is abundantly available in nature [1,2,3]. It is a long linear chain polysaccharide composed of *β*-(1-4)-linked D-anhydro-glucopyranose repeating units (AGU) with the formula (C_6_H_10_O_5_)_n_ (Figure 1) where n is the number of glucose groups and thus represents the degree of polymerization [4,5]. It is present in small quantities in the cell walls of some bacteria and more abundant in plants [6].

Cellulose and its derivatives, such as microcrystalline cellulose, methylcellulose, ethylcellulose, carboxymethyl cellulose, hydroxylpropyl methyl cellulose, cellulose acetate, etc., have found wide commercial applications in the pharmaceutical, cosmetic, food, paper, textile, and engineering industries [7,8,9,10,11].

Four different polymorphs of cellulose exist, cellulose I, II, III, and IV. Cellulose l is the natural form of cellulose known as native cellulose (NC) and the most abundantly found in nature. Cellulose I can be converted to Cellulose II, also known as alpha cellulose, by either mercerization (alkali treatment) or regeneration (solubilization and subsequent recrystallization) [12]. Cellulose II is more thermodynamically stable than cellulose I. Cellulose III which is amorphous can be obtained by treatment of either cellulose I or II with amines, while Cellulose IV can be obtained by treatment of cellulose III with glycerol [13]. NC hydrolyzes in the presence of water and acid under heat and pressure and depolymerizes into small chain polymers or crystals [14]. Some components and other impurities like wax, hemicellulose, and lignin are dissolved during the hydrolytic reaction, and filtration and washing processes to obtain a pure cellulose known as alpha cellulose [15]. Alpha cellulose is a white, odorless, and tasteless powder that is insoluble in water. It is a widely used raw material in the manufacturing of propellants, paper, paperboards, fabrics, electrical cable insulators, and cellulose derivatives, among others [7,16].

Microcrystalline cellulose (MCC) is a purified, partially depolymerized cellulose derivative obtained from alpha cellulose. The most common source of commercial MCC is fibrous wood pulp [7]. MCC is the most widely used cellulose. It is used in the food industry as a stabilizer, anti-caking agent, and emulsifier [17], while in cosmetics and pharmaceuticals it is used as an abrasive, adsorbent, adhesive, anti-caking, binder, disintegrant, bulking agent, emulsion stabilizer, etc. [7,17,18,19,20].

CPH (*Theobroma cacao* L.) is a natural waste generated from the post-harvest processing of cocoa fruits into cocoa beans which constitutes about 67–76% weight of the fresh cocoa fruit [21]. CPH is rich in minerals and fibers like hemicellulose, cellulose, lignin, and pectin [22]. CPH has been utilized as a bioplastic film in food packaging [23], biofertilizers in cocoa farming [24], an energy source in electricity generation [25], animal feeds, as well as in biopharmaceutics [22]. Few studies on cellulose isolated from CPH, available in the literature, are the extraction of MCC from CPH using alkaline pretreatment combined with ultrasonication [26], and isolation of nanocrystalline cellulose (NCC) from CPH [27].

This study aims (i) at isolating and characterizing cellulose types from CPH, which can be considered as an alternative source of cellulose to the mostly used fibrous wood pulp currently used as a pharmaceutical excipient, and (ii) to assess their mechanical and disintegration properties as direct compression excipients in a metronidazole tablet.

## 2. Materials and Methods

### 2.1. Reagents

Dried cocoa pod husks (CPH), Avicel^®^ 101 (AV) (Merck, Germany)—a brand of microcrystalline cellulose, Sodium hydroxide (Guangdong Guanghua Sci-Tech Company, Ltd., Shantou, China), Hydrochloric acid (BDH Lab Supplies, Poole, UK), and Sodium hypochlorite (Tolaram Africa Enterprises, Ltd., Lagos, Nigeria). All other reagents are of analytical grade.

### 2.2. Collection and Preparation of Cocoa Pod Husk (CPH)

Dried CPH were obtained from a local farm in Ago-Iwoye, Ogun State, Nigeria in the month of September. The CPH were further sun-dried for two weeks. The dried CPH were crushed, milled into fine powder, and screened through 850 μm sieve. It was air dried and stored in a closed container.

### 2.3. Phytochemical Screening of Cocoa Pod Husk (CPH)

Qualitative phytochemical screening of CPH was carried out by standard procedures to identify major phytoconstituents [28,29]. Frothing test was used to screen saponin while Molisch Test was used for carbohydrate. Mayers reagent, Draggendorff’s reagent, and Wagner’s reagent were used to screen alkaloids. Ferric chloride solution (FeCl_3_), lead acetate solution, gelatin solution tests were used to identify the presence of tannins. The presence of flavonoids was screened by adding a few drops of dilute sodium hydroxide (NaOH) solution to the aqueous extract of CPH followed by a few drops of dilute hydrochloric acid solution (HCl). The presence of triterpenoids was screened by dissolving CPH in chloroform, followed by the addition of acetic anhydride and concentrated sulphuric acid (H_2_SO_4_).

### 2.4. Extraction of Cocoa Alpha-Cellulose (CAC) from Cocoa Pod Husk (CPH)

CAC was extracted from the prepared CPH powder by a method previously described [15]. 500 g of the CPH powder was boiled in 3 L of distilled water (dH_2_O) for 10 min. The mixture was filtered, and the residue was then treated with 3 L of 1 M HCl for 30 min at 85 °C on a magnetic stirrer. The mixture was again filtered, and the residue was magnetically stirred with 3 L of 0.5 M HCl for 30 min at 85 °C. The mixture was further filtered, but the residue was treated this time with 3 L of 1 M NaOH solution for 30 min at 85 °C under constant stirring. Subsequently, the mixture was filtered, and this step was repeated three times. The residue was bleached with 2% sodium hypochlorite (NaOCl) solution for 60 min at 95–96 °C. This step was repeated twice to obtain alpha cellulose which was washed several times with hot dH_2_O until a neutral pH of the filtrate was obtained. The CAC was then dried in an oven at 60 °C for 16 h and the weight was noted. It was milled and screened through 650 μm sieve and stored at room temperature (RT). The yield of CAC was determined as follows:

$$\frac{\mathrm{Weight}\;\mathrm{of}\;\mathrm{CAC}}{\mathrm{Weight}\;\mathrm{of}\;\mathrm{CPH}\;\mathrm{powder}}\times 100$$

$$\frac{211.5\;g}{500\;g}\times 100=42.3\%$$

### 2.5. Isolation of Cocoa Microcrystalline Cellulose (C-MCC) from Cocoa Pod Husk (CPH)

Fifty grams CAC were placed in a Pyrex glass beaker and hydrolyzed with 1.2 L of 2.5 N HCl for 15 min at 100 °C in hot water bath. The hot acid mixture was poured into cold tap water followed by vigorous stirring on a magnetic stirrer and was allowed to stand at RT overnight. The precipitate obtained was the C-MCC which was subsequently washed with dH_2_O until neutral. The precipitate was dried for 60 min in an oven at 60 °C. Thereafter, the dried C-MCC was milled with an Osterizer blender (Model 857 Williamette Industries, Bowing Green Kentucky, KY, USA) and screened through 650 μm sieve, and stored at RT in a desiccator [30]. The yield of C-MCC was determined as follows:

$$\frac{\mathrm{Weight}\;\mathrm{of}\;\text{C-MCC}}{\mathrm{Weight}\;\mathrm{of}\;\mathrm{CAC}\;\mathrm{powder}}\times 100$$

$$\frac{84.15\;g}{220\;g}\times 100=38.25\%$$

### 2.6. General Cellulose Identification Tests

#### 2.6.1. Solubility Test

Solubility test was performed by mixing 250 mg of the cellulose in 10 mL of different solvents (dH_2_O, ethanol, chloroform, concentrated HCl, and concentrated H_2_SO_4_) [31,32].

#### 2.6.2. Chemical Tests

Two chemical tests were performed as follow:

(i): 50 mg of cellulose was weighed into a test tube and 2 mL of iodine solution was added. The mixture was allowed to stand at RT for 5 min before filtration. Two drops of 66% *v*/*v* H_2_SO_4_ were added to the residue. Color change was noted and recorded [33].

(ii): 10 mg of cellulose was weighed into a watch glass and 2 mL of iodinated zinc chloride (ClIZn) solution was added and allowed to stand for some minutes. Color change was noted and recorded [34].

#### 2.6.3. Degree of Polymerization Determination

The degree of polymerization (DP) was determined by measuring the viscosity of the cellulose using a capillary viscometer at 25 °C [35]. The *DP* was calculated using Equation (1).

(1) DP=2000∗ηspec/c∗(1+0.29∗ηspec)

where, *ηspec* (*η*/*η*_0−1_) = specific viscosity (*η*/*η*_0_ is relative viscosity)—*η*_0_ is the time used for the solvent to travel from the upper mark to the lower mark, *η* is the time used for the cellulose to travel from the upper mark to the lower mark and *c* = concentration in g/L.

#### 2.6.4. Phytochemical Screening of Cocoa Alpha-Cellulose (CAC) and Cocoa Microcrystalline Cellulose (C-MCC)

The methods previously used for the phytochemical screening of the CPH were repeated for the screening of CAC and C-MCC (Section 2.3).

### 2.7. Physicochemical Properties of the Cellulose Types

#### 2.7.1. pH Determination

One gram of each cellulose powder was dispersed in 50 mL of dH_2_O for 5 min and the pH of the supernatant liquid was taken using a pocket size pH meter (3510 model, Jenway, UK). The determination was done in triplicate to ensure data reliability.

#### 2.7.2. Bulk and Tapped Density

Ten grams of each cellulose powder were placed into a 50 mL clean and dry measuring cylinder. The volume occupied without tapping was determined and noted as *V_b_*. The measuring cylinder was then tapped manually on a soft surface bench about 300 times and the volume occupied after tapping was determined and noted as *V_t_*. This was done in triplicate to ensure data reliability, and the average volume was calculated and recorded. The bulk and tap densities were determined using Equations (2) and (3), respectively:

(2) $$Bulk\;density=Weight\;of\;sample/V_{b}$$

(3) $$Tapped\;density=Weight\;of\;Sample/V_{t}$$

#### 2.7.3. True Density

The true density of each cellulose powder was determined by the pycnometer method with xylene as the displacement liquid, as previously described by Adeleye et al. [36]. A 50-mL empty pycnometer bottle was weighed with the stopper and noted as *W*_0_. This was filled with xylene to the brim and the excess was wiped off. The weight with the stopper was then noted as *W*_1_. The difference between these weights (*W*_0_–*W*_1_) was recorded as *W*_2_. 2 g of cellulose was weighed and noted as *W_3_*, before transfer into the pycnometer bottle, and excess solvent was wiped off. The bottle was weighed again with the stopper and noted as *W_4_*. The true density, *D_t_*, was calculated using Equation (4).

(4) $$D_{t}=W_{2}*W_{3}/50\left(W_{3}-W_{4}+W_{2}+W_{0}\right)$$

#### 2.7.4. Angle of Repose

The angle of repose, *θ*, was measured by the fixed height method, as described by Adeleye et al. [36]. A funnel was clamped to a retort stand with its tip 2 cm (*h*) above a graph paper placed on a flat horizontal surface. The cellulose was carefully poured until the apex of the conical pile touched the tip of the funnel. The diameter (D) of the base of the conical pile was measured and recorded. The angle of repose, *θ*, was calculated using Equation (5).

(5) tan θ=h/r

where *r* is half of (D)

The procedure was repeated thrice to ensure data reliability and the average was calculated.

#### 2.7.5. Hausner Ratio Assesses the Inter-Particle Friction in a Powder Bed Giving an Insight into Powder Flow Properties

The Hausner ratio was calculated using Equation (6)

(6) Hausner ratio=Tapped density/bulk density

#### 2.7.6. Carr’s Index

Carr’s index is a measure of the compressibility and flow properties of a powder.

Carr’s index was calculated using Equation (7)

(7) Carr’s index=(Tapped density−bulk density)/Tapped density×100% 

#### 2.7.7. Powder Porosity

The powder porosity was derived from Equation (8)

(8) $$E=1-\left(D_{b}/D_{t}\right)\times 100\;$$

where *D_b_* is the bulk density, *D_t_* is the true density, and *E* is the porosity.

#### 2.7.8. Particle Size Distribution (PSD) Measurement

Twenty grams of each type of cellulose powder was placed on the top sieve of sieves arranged in a stack in descending degree of coarseness ranging from 1.0 mm to 90 µm. The nest of sieves was subjected to agitation on a mechanical shaker (Endecotts) at RT for 15 min. The percentage weight of the powder retained on each sieve was then determined and the mean particle size (PS) was calculated [37].

#### 2.7.9. Swelling Capacity

Three grams of each type of cellulose were placed in a 50 mL measuring cylinder. Then, 20 mL of dH_2_O was added, and the cylinder was shaken vigorously at RT every 10 min for 1 h and then allowed to stand at RT for 5 h. The swelling capacity was calculated using Equation (9).

(9) $$S=\left(V_{2}-V_{1}\right)/V_{1}\times 100$$

where *S* is the % swelling capacity, *V*_2_ is the volume of the hydrated cellulose and *V*_1_ is the tapped volume of the material prior to hydration.

#### 2.7.10. Moisture Content

Moisture content was determined by the loss on drying method, as previously reported [30]. Five grams of cellulose were transferred into a tarred glass petri dish and then dried in an oven (Ketan Lab. Oven, model; 400097, Mumbai, India) at 105 °C until a constant weight was attained. The percentage moisture loss was calculated using Equation (10).

(10) $$M=\left(W_{1}-W_{a}\right)/W_{a}\times 100$$

where *M* is the % moisture loss, *W*_b_ is the weight of the cellulose before drying and *W*_a_ is the weight of the cellulose after drying.

### 2.8. Physical Characterizations of the Cellulose Types

#### 2.8.1. Scanning Electron Microscopy (SEM)

SEM was used to examine the morphology of each cellulose powder. Images were taken by Phenom Pharos Desktop SEM (Thermos Fisher Scientific, Eindhoven, The Netherlands). The sample was prepared by placing it on a sample stub and coated by quorum technologies model Q150R with 5 nm of gold. Thereafter the prepared sample was viewed in the SEM machine via NaVCam. The morphologies of the sample at different magnifications were saved on a USB stick.

#### 2.8.2. Fourier-Transform Infrared (FTIR) Spectroscopy

FTIR was used to obtain the infrared spectrum of each cellulose powder using KBr method with FTIR spectrometer (Model: 530M, Maker: Buck Scientific, Norwalk, CT, USA). The absorbance of the powders was recorded between 1000 and 4000 cm^−1^.

#### 2.8.3. X-ray Diffraction (XRD)

An XPERT-PRO diffractometer (PAN analytical, Almelo, The Netherlands) was applied to obtain XRD spectrum of the samples at 2θ between 10° and 70° using Kα Cu radiation (λ: 1.542 Ǻ) at 25 °C and 53 ± 2% relative humidity. For this analysis, all the tested samples were pulverized and spread on sample holder thoroughly to cover the whole area of the sample holder. The crystallinity of the samples was calculated from the ratio of the integrated area of XRD spectrum and crystalline peak areas, using Origin 18 software, considering Gaussian profiles for crystalline peaks, as described by Collazo-Bigliardi et al. [38].

#### 2.8.4. Differential Scanning Calorimetry (DSC) Analysis

DSC analysis was performed on DSC3 apparatus (Mettler-Toledo GmbH, Gießen, Germany). About 10 mg of the sample was weighed into a 40 μL aluminum crucible and heated between 30 °C and 250 °C at a rate of 20 °C min^−1^ under nitrogen atmosphere flow of 20 mL·min^−^^1^.

### 2.9. Tablet Formulation and Compression

Two hundred milligrams (200 mg) of metronidazole tablet were formulated by blending 200 mg (50% *w*/*w*) metronidazole (active ingredient), 100 mg (25% *w*/*w*) cellulose (CAC, C-MCC or AV–dry binder), 50 mg (12.5% *w*/*w*) corn starch (disintegrant) and 50 mg (12.5% *w*/*w*) lactose (diluent) in a planetary mixer for 5 min. The blend was directly compressed on a Carver hydraulic hand press (model 38510E, Carver Inc., Wabash, IN, USA) using three different compression pressures (56.64 Mnm^−2^, 84.96 Mnm^−2^, and 113.28 Mnm^−2^) in a 10 mm die with a flat-faced punch lubricated with 1% dispersion of magnesium stearate in 96% ethanol before each compression.

### 2.10. Tablet Evaluation

#### 2.10.1. Determination of Tablet Hardness

Tablets were selected randomly from each formulation and the force required to break the tablet at RT was determined with a Monsanto hardness tester (DKB instrument, Mumbai. Model EH 01). The reading of the pointer on the tester was noted. Ten tablets from each formulation were evaluated and the mean was recorded.

#### 2.10.2. Determination of Tablet Friability

The weight of ten tablets selected randomly from each formulation was noted (*W*_1_) and transferred into the Friability test apparatus (Shivani scientific Ind., Mumbai, India). The apparatus was operated for 4 min at 25 revolutions per minute, and then the tablets were collected, dusted, and weighed (*W*_2_). The test was performed in triplicate and the mean was recorded. Percentage weight variation (friability) was calculated using Equation (11).

(11) $$\mathrm{Percentage}\;\mathrm{friability}=\left(\frac{W_{1}-W_{2}}{W_{1}}\right)\times 100$$

#### 2.10.3. Determination of Disintegration Time of Tablets

The disintegration test was carried out in vitro using a tablet disintegration test apparatus (DBK Instruments, Mumbai, India) within 1 L of distilled water in a beaker as the disintegration medium at 37 ± 0.5 °C [19]. Three tablets from each batch were individually placed in the basket of each tube of the disintegration apparatus. The time taken for the tablets to completely break up and pass through the wire mesh was noted and recorded. Determinations were done in triplicate.

#### 2.10.4. Statistical Analysis

Data were subjected to one-way ANOVA using GraphPad Prism Software version 5.01. *p*-values < 0.05 were considered statistically significant.

## 3. Results and Discussion

### 3.1. Yield of the Cellulose Types

The yield of CAC and C-MCC pod husk powders was 42.3% *w*/*w* and 38.25% *w*/*w*, respectively. The low yield of C-MCC in this study was expected since it is a product derived from alpha cellulose hydrolysis (amorphous regions in the alpha cellulose would have been hydrolyzed and eliminated). Interestingly, the yield of CAC and C-MCC reported in this study was higher when compared with some agro-industrial wastes such as groundnut husks [39], sorghum stalks [40], cornstalk [41], papaya stem [42], rice straw [16], and wheat straw [43].

### 3.2. Identification Parameters of the Cellulose Types

A violet-blue color was observed with all three cellulose types (CAC, C-MCC, and AV) when treated with iodinated ClIZn solution, and iodine (I) and H_2_SO_4_ indicating that they are all cellulose. They are all insoluble in dH_2_O, ethanol, and chloroform but soluble in concentrated HCl and concentrated H_2_SO_4_. This also indicated that the samples were more likely cellulose. The DP of the cellulose was determined for further identification. The result indicates that CAC had a DP of 1220 while C-MCC was 336. According to the Pharmacopeia standard, MCC is defined by a typical characteristic value of DP of less than 350 glucose units [18]. This signifies that the acid hydrolysis of CAC indeed led to the synthesis of MCC.

### 3.3. Phytochemical Screening of Cocoa Pod Husk (CPH), Cocoa Alpha-Cellulose (CAC), Cocoa Microcrystalline Cellulose (C-MCC)

The phytochemical screening of CPH revealed the presence of saponins, carbohydrates, alkaloids, tannins, flavonoids, and triterpenoids which were all absent from CAC and C-MCC (Table 1). The absence of phytochemical constituents from CAC and C-MCC is an indication that the isolated cellulose is pure and free of biological activity thus making them pharmacologically inert [7], which is a desirable property of an ideal excipient.

### 3.4. Physicochemical Properties of the Cellulose Types

The physicochemical properties of CAC, C-MCC and AV are presented in Table 2.

#### 3.4.1. Organoleptic Properties of the Cellulose Types

The isolated cellulose types (CAC and C-MCC) were odorless, tasteless, and white in color. These observations are like the reference standard (AV).

#### 3.4.2. Particle Size of the Cellulose Types

Their mean particle diameter was significantly different, with CAC (282.22 μm) > C-MCC (161.32 μm) > AV (72.51 μm). The mean particle diameter of AV was higher than the average mean size of 50 μm as specified in the official compendia for avicel 101. Studies led by Rojas et al. [44] and Doelker et al. [45] also reported smaller mean PS of 50 μm and 41.9 μm for avicel 101, respectively. Because of the small mean particle size of C-MCC and AV, they will easily deform plastically because of a process known as micro-squashing thereby enhancing cohesiveness and compactibility which may be desirable in direct compression [37].

#### 3.4.3. Density Measurements of the Cellulose Types

The bulk densities of the cellulose types were significantly different, with C-MCC (0.382 g/cm^3^) > AV (0.316 g/cm^3^) > CAC (0.273 g/cm^3^). The tapped densities were also significantly different and followed a similar order like bulk densities with C-MCC (0.473 g/cm^3^) > AV (0.469 g/cm^3^) > CAC (0.337 g/cm^3^).

The true densities of cellulose types were significantly different, and in the following order: CAC (1.535 g/cm^3^) > AV (1.507 g/cm^3^) > C-MCC > (1.494 g/cm^3^).

The pH of the cellulose types ranged from 6.5–6.7 which conformed to the United States Pharmacopeia (USP)-National Formulary reference standard of 5.0–7.5 [34].

#### 3.4.4. Moisture Content of the Cellulose Types

The moisture contents of the cellulose types were significantly different with CAC (7.2%) > AV (6.6%) > C-MCC (5.8%). However, they all complied with the USP-NF specification of a maximum of 8%.

#### 3.4.5. Swelling Properties of the Cellulose Types

Swelling capacities of the cellulose types were significantly different with CAC (36.36%) > C-MCC (27.5%) > AV (11.23%). Swelling is one formulation attribute which is essential for tablet disintegration [46]. For faster tablet disintegration, CAC with the highest swelling capacity would be desirable. The swelling tendency of these cellulose types may also be utilized as fillers in wet granulation because of their ability to promote wetting of powder blend [46].

#### 3.4.6. Flow Properties of the Cellulose Types

Powder flow is an important and significant factor in pharmaceutical dosage form designs such as blending, capsule filling, movement of material in the plant, tablet compression, and scale-up operations [47]. It enhances manufacturing efficiency and effectiveness in terms of uniformity in weight, content, and mechanical properties [48]; it also reduces the risk of capping and lamination in tablet production because it prevents entrapment of air [49,50]. Carr’s index, Hausner’s ratio, and angle of repose are powder material properties that give insight into powder flow. The results of these parameters, shown in Table 2, indicate that CAC and C-MCC had Carr’s index between 16–20%, Hausner’s ratio of 1.19–1.25, and an angle of repose of 30–40° which is an indication of a fair flow whereas AV had Carr’s index between 23–35%, Hausner’s ratio of 1.25–1.5 and angle of repose > 40° which is an indication of a poor flow. So, from these results, CAC and C-MCC have better flow than AV. The PS, particle shape, and moisture content of materials are related to the flow of powder materials. As expected, AV with the smallest PS and high moisture content had the least flow due to increased cohesiveness. The poor flow of AV confirms previous published reports [44,45,51].

### 3.5. Physical Characterization of the Cellulose Types

#### 3.5.1. Scanning Electron Microscopy (SEM)

**Figure 2:**
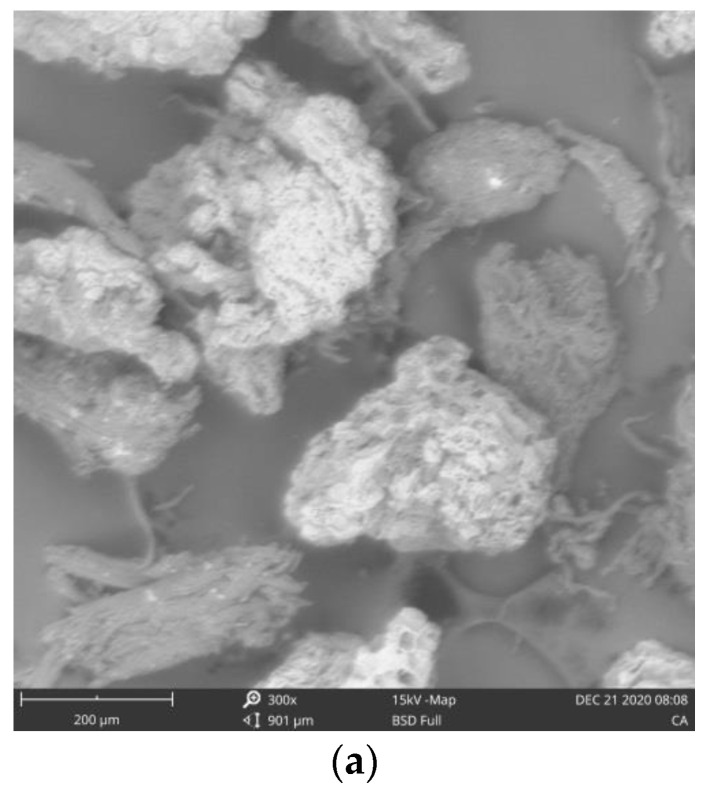

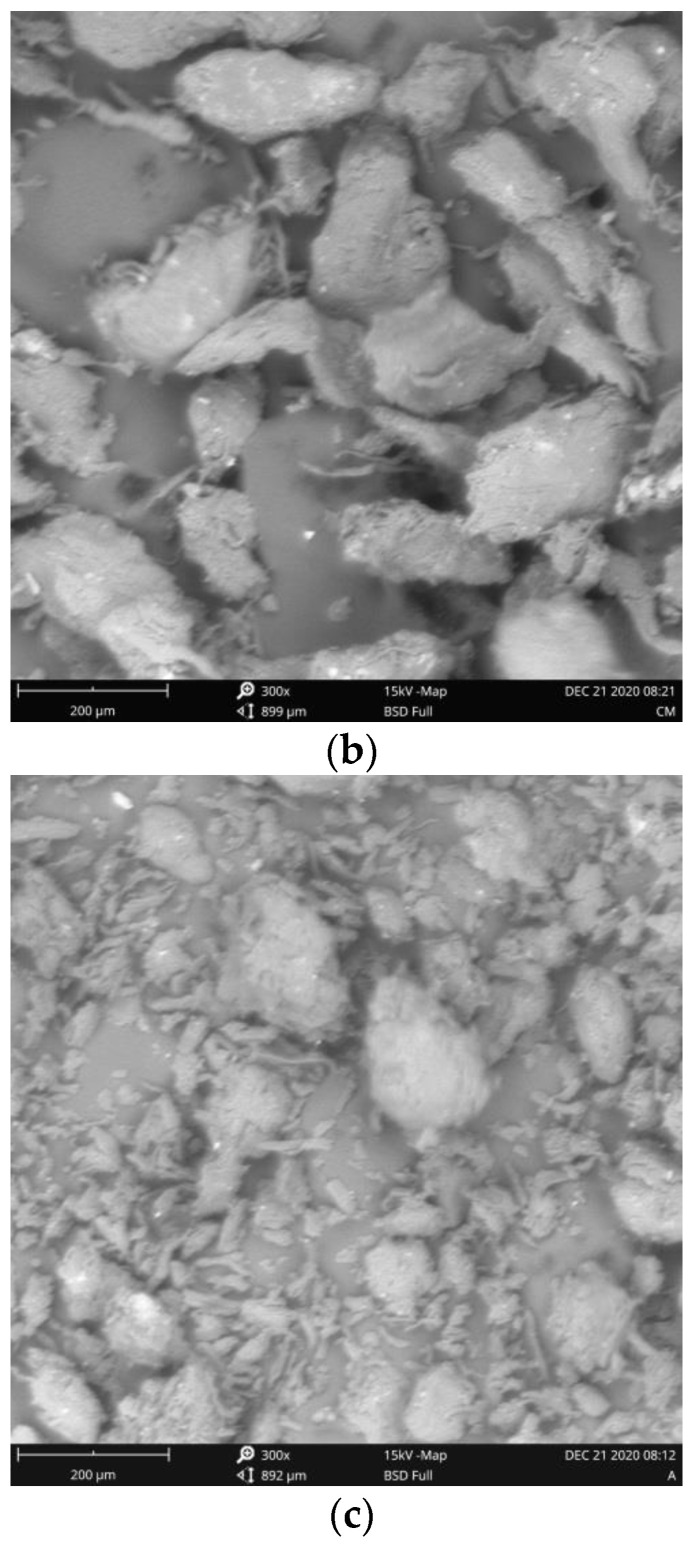
(**a**) SEM micrograph of cocoa alpha-cellulose (CAC), at 300× magnification. (**b**) SEM micrograph of cocoa microcrystalline cellulose (C-MCC) at 300× magnification. (**c**) SEM micrograph of avicel (AV) at 300× magnification.

The morphology of the cellulose types was studied by SEM and the resulting micrographs revealed that the three cellulose samples are fibrous in nature, which is typical of cellulose (Figure 2a–c) [52]. The fibers are rough and irregularly shaped. CAC has elongated and aggregated fibers while C-MCC has short and aggregated fibers, and AV has short and non-aggregated fibers. The short fibers found in C-MCC and AV are due to acid hydrolysis of alpha cellulose which led to partial depolymerization of the cellulose.

#### 3.5.2. Fourier-Transform Infrared Spectroscopy (FTIR)

**Figure 3:**
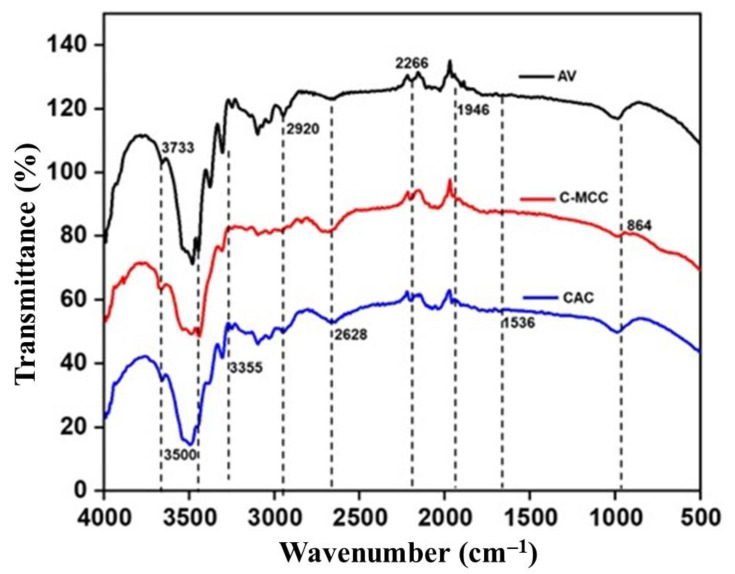
FTIR spectra of avicel (AV), cocoa microcrystalline cellulose (C-MCC) and cocoa alpha-cellulose (CAC).

FTIR spectroscopy was used to characterize the cellulose types. FTIR spectra of CAC and C-MCC, isolated from CPH, showed similarities in peak positions with differences in peak height with the reference standard—Avicel 101 (Figure 3). The similarities in peak positions indicate that CAC and C-MCC are indeed cellulose. The presence of absorption peaks at about 3733, 3500, 3492, 2920, 2826, 1946, 1536, 1372, and 864 cm^−1^ were associated with the representative functional groups of cellulose [53,54]. Noticeably, the broad absorption peak at 3492 cm^−1^ for AV had slightly shifted to 3500 cm^−1^ for C-MCC and, CAC suggesting the increasingly exposed cellulose region through the treatments. Furthermore, the absorption peak at 864 cm^−1^ corresponds to the blending vibrations in C-OH groups [55]. Peaks in the 1500–2000 range are C=C in plane aromatic stretching vibrations. Peaks at 1536 cm^−1^ correspond to C-H bending (aromatic compounds) or C=C=C stretching (alkene). Peaks at 2628 cm^−1^ correspond to symmetric C-H stretching. Peaks at 3733.60 cm^−1^ correspond to O-H stretching vibrations for free OH. The broad band of the unassigned characteristics peaks at 3300–3400 cm^−1^ for the cellulose is due to inter and intra molecular O–H stretching vibrations which is characteristic of cellulose. A peak at 1936 cm^−1^ corresponds to O–H bending vibration of water adsorbed in cellulose and some hemicellulose. CH_2_ bending known as the crystallinity band is present in the cellulose at 1400–1315 cm^−1^ peak [56]. The presence of C–O–C ether stretching at a peak of 1257 cm^−1^ for CAC and C-MCC is an indication that the bleaching treatment used during the isolation of the cellulose did not effectively remove lignin from the cellulose. The presence of C–O–C pyranose stretching skeletal vibration in all the spectra confirms the presence of cellulose [55].

#### 3.5.3. X-ray Diffraction (XRD)

**Figure 4:**
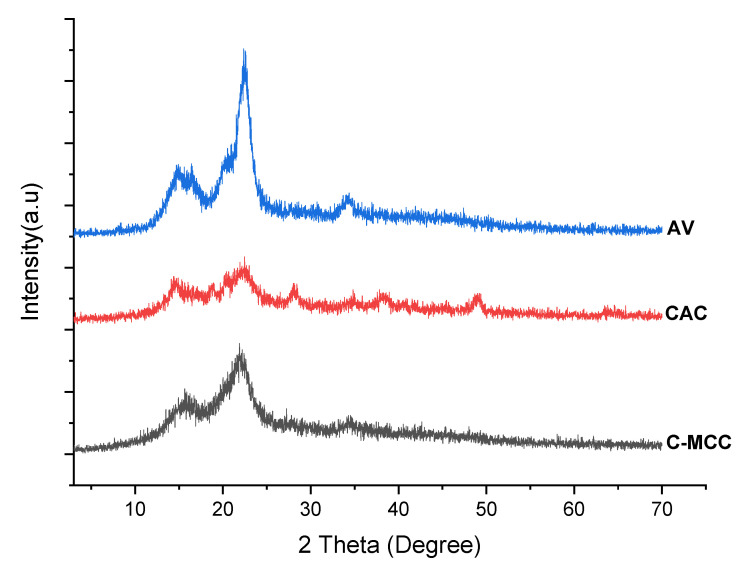
XRD patterns of avicel (AV), cocoa alpha-cellulose (CAC) and cocoa microcrystalline cellulose (C-MCC).

Cellulose is a semi-crystalline polysaccharide which includes crystalline and amorphous regions [57]. There are different crystallite forms of cellulose which are C_I_, C_II_, C_III_, and C_IV_ [12]. Cellulose I is native cellulose and the most abundantly found in nature. Cellulose II can be prepared by either mercerization (alkali treatment) or regeneration (solubilization and subsequent recrystallization) [12]. Figure 4 displays the XRD spectrum of the C-MCC and CAC isolated from cocoa pod husk and AV cellulose used as standard. In the C-MCC sample, the typical crystalline peaks of cellulose 004, 200, and 002 were observed at 2θ: 15–16°, 22°, and 34° as described in previous literature [58,59,60]. However, the characteristic peak of C-MCC is broader compared to AV. This peak broadening may be due to an increase in the amorphous nature of C-MCC during the synthesis process. CAC shows only one broader peak at 2θ: 19.0–25.0° presumably due to the high amorphous nature of the synthesized cellulose. From the calculation, AV showed the highest crystallinity index (69.26%), followed by C-MCC (43.83%), and lastly by CAC (26.32%). The lowermost crystallinity for CAC was possibly related to the alkaline treatment that softened the structure of cellulose via the swelling process. The result of the swelling capacities of the cellulose is also confirmed by the crystallinity index result. CAC with the highest swelling capacity had more amorphous regions which are responsible for water uptake and thus swelling [61]. The results of the XRD and FTIR of different forms of cellulose agreed with the reported literature since both analyses confirmed the presence of cellulose.

#### 3.5.4. Differential Scanning Calorimetry (DSC)

**Figure 5:**
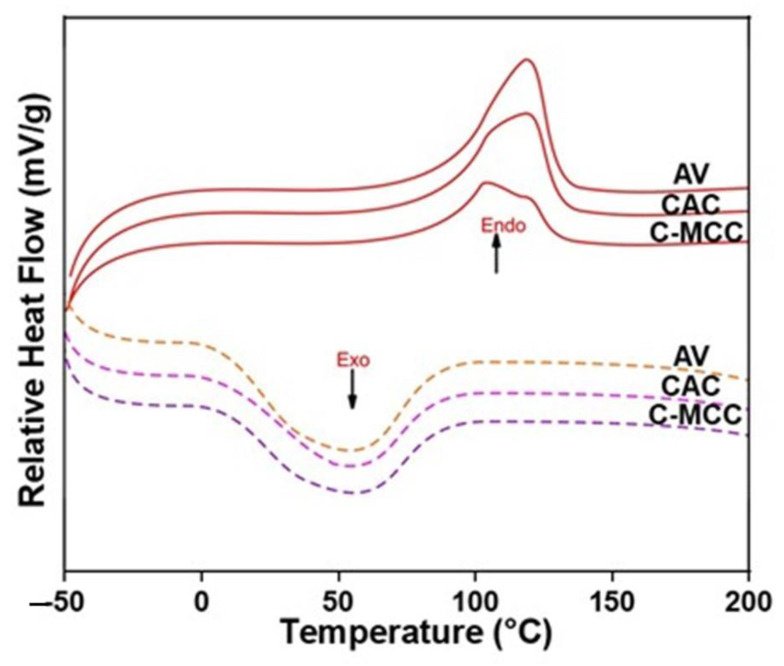
DSC curves of avicel (AV), cocoa alpha-cellulose (CAC) and cocoa microcrystalline cellulose (C-MCC).

DSC analysis was used to evaluate the energy consumption properties and thermal stability of the cellulose types (Figure 5). The temperature of 30–200 °C was selected since temperatures above 300 °C could cause decarboxylation reactions and depolymerization of cellulose. The thermal stability of cellulose depends upon the degree of crystallinity and the type and/or source of the cellulose. The broad endothermic peak characteristic of alpha cellulose was observed in the range of 90–130 °C due to the evaporation of moisture and other volatile components. The shift in the maximum temperature of dehydration/evaporation to higher values in the case of AV and CAC may be due to the decrease in their structural crystallinity index. This result is consistent with previous studies on cellulose extracted from different sources [62,63]. The glass transition temperature (Tg) peak of CAC was 68 °C which is within the glass transition temperature of amorphous cellulose (60–70 °C). The possible reason for the reduction of Tg may be due to an increase in the molecular mobility due to the disruption of hydrogen bonds in adjacent macro molecules leading to a decrease in Tg of the amorphous regions inducing a transformation from a hard and rigid to soft and flexible state. The glass transition peak of the amorphous region observed with CAC was absent in C-MCC and AV [64]. The disappearance of this peak in these samples suggests that C-MCC and AV are more crystalline and higher in purity compared to CAC. The observed exothermic peaks around 50 °C in C-MCC, CAC, and AV may be attributed to charring. As a result of various treatment methods, cellulose crystallites can undergo reorientation and rearrangement, leading to a more compact crystal structure and hence, improved thermal stability as observed with C-MCC and AV [65,66].

#### 3.5.5. Evaluation of Tablets

The isolated cellulose types were employed at 25% concentration as a direct compression excipient in the formulation of a metronidazole tablet. The mechanical and disintegration properties of the tablets were assessed to check their suitability in tablet formulation as an excipient. The hardness, friability, and disintegration time of the formulated tablets are presented in Table 3. Tablet hardness is a property that ensures the appropriateness of tablet strength. In this study, there was generally a significant increase in the hardness of the tablets (<0.0001) with an increase in compression pressure due to an increase in inter-particulate bonding. The British Pharmacopeia specifies that the hardness of a conventional uncoated tablet is 4–10 kg/F [67]. None of the tablets formulated with CAC passed the test while all the tablets formulated with C-MCC and AV passed. The reason for the failure could be attributed to the poor thermoplastic properties of the cellulose influenced by the presence of some quantity of hemicellulose and lignin [68]. Suzuki and Nakagami [69] reported reduced crushing strength as crystallinity decreased while Ling et al. [70] reported amorphous materials to have less tensile strength. A similar trend was observed in this study with CAC having the least crystallinity and the smallest hardness. The friability—a measure which describes the tendency of a tablet to break or crumble during manufacturing, packaging, and transportation—is often related to hardness. The friability of the tablets increased with an increase in compression pressure although the increase was not significantly different. However, the tablets formulated with CAC and formulation F4 failed the friability test since their values were greater than 1% while formulations F5–F9 passed. Friability is often inversely related to hardness. As expected, as the hardness of the tablets increased, friability decreased. Disintegration time increased with an increase in compression pressure. AV formulation had a higher disintegration time compared to C-MCC formulations. However, both formulations complied with BP specifications. CAC did not produce tablets with satisfactory properties in terms of hardness and friability but shows a promising activity when formulating a fast-disintegrating conventional formulation or orally disintegrating formulations because of its high swelling capacity.

## 4. Conclusions

The mechanical and disintegration properties of metronidazole tablets formulated by direct compression using C-MCC or AV complied with pharmacopeia specifications. C-MCC possessed some fundamental characteristics suitable to make it a pharmaceutical excipient comparable to AV in varieties of pharmaceutical processes and applications.

## Figures and Tables

**Table 1:** Phytochemical Screening of Cocoa Pod Husk (CPH), Cocoa Alpha-Cellulose (CAC), Cocoa Microcrystalline Cellulose (C-MCC).

Constituent	CPH	CAC	C-MCC
Saponin	+	-	-
Tannins	+	-	-
Alkaloids	+	-	-
Carbohydrate	+	-	-
Flavonoids	+	-	-
Triterpenoids	+	-	-

+ = present, - = absent.

**Table 2:** Physicochemical properties of the cellulose types. Cocoa alpha-cellulose (CAC) and cocoa microcrystalline cellulose (C-MCC) were compared with avicel (AV).

Parameters	CAC	C-MCC	AV
Color	Off white	White	White
Odor	Odorless	Odorless	Odorless
Mean particle diameter (μm)	282.22 ± 0.12	161.32 ± 0.04	72.51 ± 0.53
True density (g/cm^3^)	1.535 ± 0.21	1.494 ± 0.13	1.507 ± 0.35
Bulk density (g/cm^3^)	0.273 ± 0.02	0.382 ± 0.06	0.316 ± 0.02
Tapped density (g/cm^3^)	0.337 ± 0.01	0.473 ± 0.04	0.469 ± 0.10
Carr’s Index (%)	18.93	19.11	32.62
Hausner’s ratio	1.23	1.24	1.48
Angle of repose (°)	35.05 ± 0.54	31.09 ± 0.33	44.29 ± 0.61
Swelling Capacity (%)	36.36 ± 0.05	27.5 ± 0.01	11.23 ± 0.08
Moisture Content (%)	7.20 ± 0.62	5.8 ± 0.37	6.6 ± 0.60
pH	6.7 ± 0.37	6.5 ± 0.14	6.5 ± 0.21
Crystallinity Index (%)	26.32	43.83	69.26

**Table 3:** Tablet hardness, friability, and disintegration time.

Formulation Code	Hardness (kg/F)	Friability %	Disintegration (min)
F1	2.8 ± 3.2	2.84 ± 0.03	0.82 ± 0.20
F2	2.5 ± 3.4	2.12 ± 0.02	1.15 ± 0.14
F3	3.1 ± 2.1	1.60 ± 0.04	1.20 ± 0.17
F4	3.8 ± 2.7	1.25 ± 0.05	4.12 ± 0.38
F5	4.7 ± 3.2	0.85 ± 0.01	4.92 ± 0.25
F6	5.4 ± 2.0	0.68 ± 0.06	5.21 ± 0.44
F7	5.8 ± 2.6	0.60 ± 0.01	5.70 ± 0.32
F8	7.1 ± 3.0	0.48 ± 0.02	6.55 ± 0.30
F9	8.7 ± 2.1	0.32 ± 010	8.24 ± 0.56

F1 = CAC at 56.64 Mnm^−2^ compression pressure, F2 = CAC at 84.96 Mnm^−2^ compression pressure, F3 = CAC at 113.28 Mnm^−2^ compression pressure, F4 = C-MCC at 56.64 Mnm^−2^ compression pressure, F5 = C-MCC at 84.96 Mnm^−2^ compression pressure, F6 = C-MCC at 113.28 Mnm^−2^ compression pressure, F7 = AV at 56.64 Mnm^−2^ compression pressure, F8 = AV at 84.96 Mnm^−2^ compression pressure, F9—AV at 113.28 Mnm^−2^ compression pressure.

## Data Availability

The data can be requested from the corresponding authors.
